# Different Ligands of the TRPV3 Cation Channel Cause Distinct Conformational Changes as Revealed by Intrinsic Tryptophan Fluorescence Quenching*

**DOI:** 10.1074/jbc.M114.628925

**Published:** 2015-03-31

**Authors:** Bert Billen, Marijke Brams, Sarah Debaveye, Alina Remeeva, Yeranddy A. Alpizar, Etienne Waelkens, Mohamed Kreir, Andrea Brüggemann, Karel Talavera, Bernd Nilius, Thomas Voets, Chris Ulens

**Affiliations:** From the ‡ Laboratory of Structural Neurobiology and TRP Research Platform Leuven (TRPLe), Department of Cellular and Molecular Medicine, University of Leuven, Herestraat 49 Box 601, 3000 Leuven, Belgium,; the § Laboratory of Ion Channel Research and TRP Research Platform Leuven (TRPLe), Department of Cellular and Molecular Medicine, University of Leuven, Herestraat 49 Box 802, 3000 Leuven, Belgium,; the ¶ Laboratory of Protein Phosphorylation and Proteomics, Department of Cellular and Molecular Medicine, University of Leuven, Herestraat 49 Box 901, 3000 Leuven, Belgium, and; ‖ Nanion Technologies GmbH, Gabrielenstrasse 9, D-80636 Munich, Germany

**Keywords:** fluorescence, membrane protein, membrane reconstitution, protein purification, transient receptor potential channels (TRP channels), camphor, human TRPV3, icilin, quenching

## Abstract

TRPV3 is a thermosensitive ion channel primarily expressed in epithelial tissues of the skin, nose, and tongue. The channel has been implicated in environmental thermosensation, hyperalgesia in inflamed tissues, skin sensitization, and hair growth. Although transient receptor potential (TRP) channel research has vastly increased our understanding of the physiological mechanisms of nociception and thermosensation, the molecular mechanics of these ion channels are still largely elusive. In order to better comprehend the functional properties and the mechanism of action in TRP channels, high-resolution three-dimensional structures are indispensable, because they will yield the necessary insights into architectural intimacies at the atomic level. However, structural studies of membrane proteins are currently hampered by difficulties in protein purification and in establishing suitable crystallization conditions. In this report, we present a novel protocol for the purification of membrane proteins, which takes advantage of a C-terminal GFP fusion. Using this protocol, we purified human TRPV3. We show that the purified protein is a fully functional ion channel with properties akin to the native channel using planar patch clamp on reconstituted channels and intrinsic tryptophan fluorescence spectroscopy. Using intrinsic tryptophan fluorescence spectroscopy, we reveal clear distinctions in the molecular interaction of different ligands with the channel. Altogether, this study provides powerful tools to broaden our understanding of ligand interaction with TRPV channels, and the availability of purified human TRPV3 opens up perspectives for further structural and functional studies.

## Teaser

**Background:** Further insight into the structural biology of TRP channels is crucial to explain molecular mechanisms of channel function.

**Results:** We purified TRPV3, demonstrated its functional integrity, and used fluorescence spectroscopy to study ligand binding.

**Conclusion:** TRPV3 ligands induce different conformational changes as observed by tryptophan fluorescence quenching.

**Significance:** Availability of purified TRPV3 allows functional assays outside the cellular context and facilitates future structural studies.

## Introduction

Transient receptor potential (TRP)^2^ channels constitute a large family of transmembrane proteins that form tetrameric cation-permeable channels (1). TRPV3 is a member of the TRP vanilloid (TRPV) family and belongs to the temperature-sensitive TRP channels (so-called “thermo-TRPs”), showing strong activation by warming in the thermal range of 33–39 °C. The channel is expressed robustly in keratinocytes of the skin, tongue, and nose and is also present in peripheral sensory neurons in humans (2–4). Because of this expression pattern and temperature sensitivity, TRPV3 was proposed as a putative thermosensor. However, it is still unclear at present whether TRPV3 is in fact involved in acute thermal transduction. Thermosensation is thought to be directly mediated by sensory neurons of the dorsal root ganglia that terminate as free nerve endings in the skin (5–7). Initial studies reported that TRPV3-deficient mice exhibit clear behavioral deficits in warmth sensation (8). However, TRPV3 channels are not expressed in dorsal root ganglion neurons in mice, and more recent studies debate the question of whether TRPV3 plays a direct role in thermosensation (9–11). TRPV3 mRNA is also found throughout the brain, but its function here remains unknown (3, 4). Some studies have indicated TRPV3 to be involved in emotional regulation and synaptic plasticity (12–16).

In addition to its temperature sensitivity, TRPV3 is also responsive to a number of exogenous ligands, including plant-derived terpenoids like camphor, menthol, and eucalyptol (17–19). Although it is tempting to argue that TRPV3 might be involved in the anesthetic, analgesic, and antipruritic properties of these compounds in over-the-counter therapeutic products, some caution should be taken, because most of these ligands are quite promiscuous in their interactions with thermo-TRPs. For instance, the popular cooling agent menthol activates TRPV3, but also TRPM8 and TRPA1, two cold-activated thermo-TRPs (20, 21). At room temperature, menthol application results in a cooling effect, whereas in warmer environments, it produces a warm sensation. It was hypothesized that the cold perception may be mediated by TRPM8 and the warm perception by TRPV3 (21). Icilin, another TRPM8 and TRPA1 agonist and supercooling agent was found to inhibit TRPV3, which might contribute to the strong cooling perception of the compound (22).

Until recently, our understanding of TRP channel structural biology was limited to either high-resolution (up to 1.6 Å) x-ray structures from cytoplasmic domains of TRP channels or low-resolution (up to 19 Å) electron microscopy (EM) studies of integral channels (23–30). In a recent publication, the three-dimensional structure of rat TRPV1 was solved by cryo-EM with a resolution of at best 3.4 Å (31, 32). Although the reported TRPV1 structures in the presence and absence of pharmacological probes suggest fundamental differences in channel gating between TRP channels and typical voltage-gated channels, the structures still lack sufficient atomic detail to answer fundamental questions like how and where vanilloid ligands bind to the channel or to explain molecular mechanisms of gating in TRP channels. Comparison of the TRPV1 EM structure with low-resolution EM structures from TRPV2 and TRPV4 reveals a shared global architecture within the TRPV family (30, 33). It is, however, clear that high-resolution structures of individual full-length TRPV channels will be indispensable leads for new testable hypotheses to elucidate the molecular frameworks that underlie the gating characteristics of specific TRPV channels. In particular, TRPV3 displays some unusual channel properties within the vanilloid receptor family (16). For example, TRPV3 exhibits a strong sensitization upon repeated short term exposure to heat or chemical agonists, in contrast with other TRPV channels that desensitize upon repeated activation (2, 4, 34–36). Moreover, TRPV3 displays an unusually large unitary conductance (34) and is potentiated by hydrolysis of the membrane lipid phosphatidylinositol 4,5-bisphosphate rather than inhibited like most other TRPV channels (37).

In our aim to study molecular mechanisms underlying the function of TRPV channels, we devised a method for biochemical purification of full-length human TRPV3 for structural studies. We demonstrate that the detergent-purified protein is a functional channel. While crystallization efforts for x-ray diffraction are currently ongoing, we use detergent-purified TRPV3 to study ligand binding in fluorescence spectroscopy experiments.

## EXPERIMENTAL PROCEDURES

### 

#### 

##### Protein Expression

For expression in Sf9 insect cells (*Spodoptera frugiperda*), cDNA encoding human TRPV3 (hTRPV3) was subcloned into the pFastBac vector, and baculovirus was produced according to the manufacturer's protocol (Bac-to-Bac, Invitrogen). The hTRPV3 construct was expressed as a fusion protein with a PreScission cleavage site, C-terminal green fluorescent protein (GFP), and His_8_ tag. Sf9 cells were harvested 60 h postinfection by centrifugation (10,000 × *g* for 20 min) and resuspended in buffer A (200 mm NaCl, 50 mm Tris, pH 7.5), supplemented with 10 μg·ml^−1^ DNase, 5 mm MgCl_2_, and protease inhibitors (1 mm phenylmethanesulfonyl fluoride, 1 μg·ml^−1^ pepstatin, 1 μg·ml^−1^ leupeptin, and 1 μg·ml^−1^ aprotinin). The resuspended cells were subsequently lysed by sonication and centrifuged (10,000 × *g* for 20 min) to discard unbroken cells. Membranes were harvested by ultracentrifugation (125,000 × *g* for 1 h) and resuspended in buffer A, supplemented with protease inhibitors (1 ml of buffer/g of membrane). Homogenized membranes were sampled in 360-μl aliquots, snap-frozen in liquid N_2_, and stored at −80 °C until further use.

##### Detergent Screen

Isolated crude membrane aliquots were thawed on ice, and detergent stock was added to a final concentration of 1 or 2% (w/v), depending on the critical micelle concentration (CMC), with a final concentration at ≥3 × CMC. In the screen, most commonly used detergents, including *n*-decyl-β-d-maltopyranoside (DM), *n*-undecyl-β-d-maltopyranoside, *n*-dodecyl-β-d-maltopyranoside (DDM), CHAPS, *n*-octyl-β-d-glucopyranoside, lauryl maltose neopentyl glycol, *n*-dodecyl-*N*,*N*-dimethylamine-*N*-oxide (LDAO), CHAPSO, *n*-decylphosphocholine (Fos-10), *n*-dodecylphosphocholine (Fos-12), and *n*-tetradecylphosphocholine (Fos-14) were tested (anagrade, Anatrace). Membrane-detergent mixtures were rotated for 1 h at 4 °C for solubilization. Insoluble parts were removed by ultracentrifugation (1 h at 60,000 × *g*), and 100 μl of clear supernatant was injected on a gel filtration column (Superose 6 10/300 GL, AKTA purifier system, GE Healthcare) coupled to a fluorescence detector (RF-10AXL, Shimadzu). The running buffer consisted of buffer A, supplemented with the respective detergent at a concentration of ∼1.5 × CMC.

##### Protein Purification

Expression and isolation of crude membranes were performed as described above. For solubilization, 2% (w/v) DDM and 0.2% (w/v) CHAPS (anagrade, Affymetrix) were added to gently thawed membranes, and the sample was stirred for 1 h at 4 °C. The solubilizate was ultracentrifuged (60,000 × *g* for 1 h) to discard non-solubilized material and protein aggregates. The supernatant was incubated for 30 min with GPF-nanobody-coupled agarose beads (GFP-Trap_A, Chromotek) at 4 °C. Then the flow-through was discarded, and the beads were washed with 10 column volumes of buffer A, supplemented with 0.2% (w/v) DDM and 0.2% (w/v) CHAPS. To elute hTRPV3, the fusion protein was cleaved off overnight by incubation with PreScission protease (0.3 mg·ml^−1^) at 4 °C, leaving the GFP-His tag bound to the beads. The total elution (eluate + 5 column volumes of wash) was then concentrated to ∼1 ml (100 kDa cut-off, Vivaspin 20, Sartorius) and loaded on a Superose 6 10/300 GL gel filtration column (AKTA purifier system, GE Healthcare). The running buffer consisted of buffer B (100 mm NaCl, 10 mm Tris, pH 7.5), supplemented with 0.03% (w/v) DDM and 0.1% (w/v) CHAPS. After analysis on SDS-PAGE, the peak fractions were compiled and concentrated (100 kDa cut-off, Vivaspin 6, Sartorius).

##### Fluorescence Detection Size Exclusion Chromatography (FSEC)-based Thermostability Assay

Isolated crude membrane aliquots were solubilized in the presence of 2% (w/v) DDM. To determine the melting temperature (temperature of half-maximal fluorescence amplitude), aliquots of solubilized membranes were each heated for 10 min to a different temperature in the range of 4–70 °C. The aliquots were then centrifuged for 20 min at 82,000 × *g*, and the supernatants were consecutively injected on a gel filtration column (Superose 6 10/300 GL, AKTA purifier system, GE Healthcare) coupled to a fluorescence detector (RF-10AXL, Shimadzu). A melting curve was constructed by plotting the relative fluorescence signal of the hTRPV3 peaks (with 4 °C set as 1) against the corresponding temperatures. The data were fit with a sigmoidal curve using nonlinear regression in Prism 6 (GraphPad). To test the effect of additives on hTRPV3 thermostability, crude membranes were preincubated at 4 °C for 60 min with a variety of salts, lipids, detergents, and ligands before heating to 40 °C for 10 min. This heating temperature was set slightly higher than the calculated melting temperature for apo-hTRPV3 (36 °C), in order to better monitor the potential stabilizing effects of additives. Detergent additives 0.1–1% (w/v) CHAPS, 0.15% (v/v) 6-cyclohexyl-1-hexyl-β-d-maltoside (Cymal-6), 0.15% (v/v) 7-cyclohexyl-1-heptyl-β-d-maltoside (Cymal-7), 0.2% (v/v) *n*-octyl-β-d-glucoside, 0.02% (v/v) *n*-undecyl-β-d-maltopyranoside, 0.02% (v/v) LDAO, 0.02% (v/v) CHAPSO, 0.2% (v/v) *n*-decyl-*N*,*N*-dimethyl-3-ammonio-1-propanesulfonate (zwittergent 3-10), and 0.2% (v/v) *n*-tetradecyl-*N*,*N*-dimethyl-3-ammonio-1-propanesulfonate (zwittergent 3-14) were purchased in anagrade quality from Anatrace. Lipid additives 0.2 mm 1-hexadecanoyl-2-octadecenoyl-glycero-3-phosphocholine; 0.2 mm 1-palmitoyl-2-oleoyl-glycero-3-phosphoethanolamine; 0.2 mm 1-palmitoyl-2-oleoyl-glycero-3-phospho-(1′-*rac*-glycerol); 0.2 mm 1-palmitoyl-2-oleoyl-glycero-3-phospho-l-serine, sodium salt; 0.2 mm 1,2-dioctadecenoyl-glycero-3-phospho-l-serine, sodium salt; 0.2 mm 1,2-dioctadecenoyl)-glycero-3-phosphocholine; 0.2 mm 1,2-dioctadecenoyl)-glycero-3-phosphoethanolamine; 0.2 mm 1,2-ditetradecanoyl-glycero-3-phosphocholine; 0.2 mm 1,2-dihexadecanoyl-glycero-3-phosphocholine; 0.2 mm 1,2-diheptanoyl-glycero-3-phosphocholine; 0.2 mm 3β-hydroxy-5-cholestene-3-hemisuccinate; 0.2 mm 1′,3′-bis[1,2-dimyristoylglycero-3-phospho]-glycerol, sodium salt (cardiolipin); 0.2 mm d-*erythro*-sphingosylphosphorylcholine (sphingomyelin, porcine brain); 0.2 mm *N*-octadecanoyl-d-*erythro*-sphingosine (ceramide, porcine brain); and 0.2 mm 5,8,11,14-*cis*-eicosatetraenoylethanolamide (anandamide) were purchased from Avanti Lipids. Other tested additives 0.1% (v/v) soybean oil, 1–100 mm CaCl_2_, 100 mm MgCl_2_, 100 mm ZnCl_2_, 3 mm menthol, 5 mm camphor, 5 mm carvacrol, 1 mm 2-aminoethoxy-diphenyl borate (2-APB), and 0.1 mm icilin were purchased from Sigma-Aldrich. After heating, the samples were centrifuged (20 min at 82,000 × *g*), and the supernatants were consecutively injected on a gel filtration column (Superose 6 10/300 GL, AKTA purifier system, GE Healthcare) coupled to a fluorescence detector (RF-10AXL, Shimadzu). For comparison, the relative fluorescence signals of preincubated samples are shown in a bar plot, together with the relative fluorescence signal from non-pretreated samples at 4 and 40 °C (with the 4 °C set as 1).

##### Microfluorimetric Intracellular Ca^2+^ Imaging Experiments

Sf9 cells were infected with baculovirus 36–48 h before the imaging experiments and incubated at 28 °C. Immediately prior to the experiment, the cells were incubated with 2 μm Fura-2/AM ester for 30 min at 28 °C. Fluorescence images were acquired at room temperature (21–26 °C) on a Cell[caret]M system (Olympus). The fluorescence intensity of individual cells was measured at excitation wavelengths of 340 and 380 nm and monitored as a ratio of the fluorescence at both wavelengths (*F*_340_/*F*_380_) after correction for the background fluorescence. Throughout the experiments, cells were perfused with standard Krebs solution containing 150 mm NaCl, 6 mm KCl, 10 mm HEPES, 1.5 mm CaCl_2_, 1 mm MgCl_2_, 10 mm glucose monohydrate, pH-adjusted to 7.4. The data were classified semiautomatically using MATLAB (MathWorks) and analyzed with Origin version 7.0 (OriginLab).

##### Patch Clamp Recordings on Planar Lipid Bilayers

We reconstituted detergent-purified hTRPV3 in lipid bilayers and assayed its functional properties using the planar patch clamp technique. The lipid bilayers were obtained from giant unilamellar vesicles. Giant unilamellar vesicles were freshly prepared by electroformation (Vesicle Prep Pro, Nanion Technologies) with 10 mm diphyntanoylphosphatidylcholine (Avanti Lipids) and 1 mm cholesterol (Avanti Lipids). Lipid bilayers were then formed by adding giant unilamellar vesicles directly into the Port-a-Patch recording chamber (Nanion Technologies). After seal formation (>5 gigaohms), ramp and constant voltage protocols were applied for 5–10 min to monitor seal stability and exclude the presence of contaminations. DDM-purified hTRPV3 (0.2–1 μl of 0.5 μg·ml^−1^) was then added to the planar lipid bilayer and incubated for 5 min. First, a ramp voltage protocol was applied (−100 to 100 mV in 2 s) to assess channel activity. Then single-channel recordings were performed at constant holding potentials (different voltages). To exclude possible ligand- or vehicle-induced artifacts, we tested all ligands (2-APB, camphor, menthol, icilin, and eucalyptol) in control experiments on mock bilayers (no hTRPV3 present). All current recordings were terminated upon the addition of ruthenium red to verify current block. Data were recorded at room temperature (21–26 °C). The recording solutions contained 200 mm KCl, 10 mm HEPES, pH 7.0 (internal) and 200 mm NaCl, 10 mm HEPES, pH 7.0 (external). The data were recorded at a sampling rate of 20 kHz, low pass-filtered at 2 kHz (HEKA amplifier), and analyzed with Clampfit version 10 (Molecular Devices) and Prism version 6 (GraphPad).

##### Intrinsic Tryptophan Fluorescence Quenching Assay

Fluorescence quenching experiments were performed at room temperature (21–26 °C) using a FlexStation 3 microplate reader (Molecular Devices). Seven mutant channels (W433Y, W481Y, W493Y, W521Y, W559Y, W692Y, and W710Y) were synthesized using a QuikChange strategy (Stratagene) and verified by sequencing (LGC Genomics). The mutants were expressed and purified as described above. Freshly purified wild type or mutant hTRPV3 (∼1 mg·ml^−1^) was excited at 295 nm, and emission spectra were recorded between 320 and 370 nm. To correct for background signal, control spectra were recorded from separate wells containing buffer + DMSO + ligand. The fluorescence peak for wild type hTRPV3 was observed near 336 nm, which was used as the emission wavelength for calculating the quenching plots. The quenched fluorescence was plotted as *F*/*F*_0_, where *F*_0_ and *F* are the fluorescence peak amplitude in the absence and presence of ligand, respectively. Lysozyme (∼1 mg·ml^−1^) was used in control experiments to evaluate for potential nonspecific quenching effects. To calculate *K_D_* values, quenched fractions (1 − *F*/*F*_0_) were plotted against concentration and fit with nonlinear regression using Prism version 6 (GraphPad). Fluorescence quenching experiments were performed in the absence or presence of 0.1–3 mm menthol, 0.1–10 mm camphor, 0.1–10 mm 1,8-cineole (eucalyptol), 0.1–10 mm 2-APB, or 0.01–1 mm icilin. All ligands were freshly diluted to a final concentration from frozen stock solutions in DMSO. Because of poor solubility, aqueous solutions with concentrations higher than 10 mm camphor or 3 mm menthol could not be completely dissolved. All data represent means ± S.E. Statistical comparisons were made using an unpaired Student's *t* test.

## RESULTS

### 

#### 

##### Protein Expression and Purification

**FIGURE 1.**
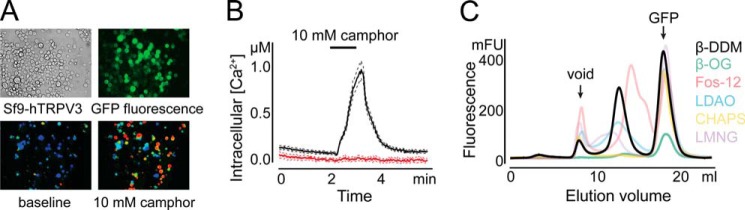
**Expression and detergent screen of human TRPV3.** *A*, Sf9 cells infected with recombinant baculovirus (*top left*), exposed to blue light to monitor GFP fluorescence (*top right*), and in the absence and presence of 10 mm camphor during a calcium imaging experiment (*bottom left* and *right*, respectively). *B*, calcium imaging experiment, showing the response of Sf9 cells expressing hTRPV3-GFP (*red*) and control (*black*) to 10 mm camphor. The *dashed lines* represent mean ± S.E. (*n* = 52). *C*, detergent screen of hTRPV3. The graph shows a superposition of FSEC profiles from detergent-solubilized hTRPV3-GFP. Comparison of peak amplitude and symmetry between different detergents reveals the superior extraction efficiency and stability of hTRPV3-GFP in DDM. *OG*, lauryl maltose neopentyl glycol; *LMNG*, *n*-octyl-β-d-glucopyranoside.

Human TRPV3 (hTRPV3) was expressed in Sf9 insect cells as a fusion with C-terminal GFP. To evaluate the effect of the GFP fusion on the functional integrity of hTRPV3, we performed calcium imaging experiments (Fig. 1, *A* and *B*). When insect cells were challenged with camphor, a TRPV3 agonist, the internal calcium concentration increased reversibly in cells that exhibit GFP fluorescence. In contrast, non-infected cells (no GFP fluorescence) remained unresponsive to the ligand. These results indicate that the channel is functional and suggest that the GFP fusion does not impede channel hTRPV3 activity.

Extraction of a membrane protein from the membrane into an aqueous solution requires the use of detergents, which shield the hydrophobic surface of the protein. However, most detergent-solubilized membrane proteins tend to aggregate or even denature upon solubilization. Therefore, the choice of detergent is critical for maintaining the protein solubilized in its native oligomeric state. In order to find suitable buffer conditions to solubilize hTRPV3, we set up a broad detergent screen. We employed the FSEC assay (38) to rapidly analyze the effects of detergents on analytical samples of crude membranes. Fig. 1*C* shows a selection of the 11 tested detergents. DM, DDM, LDAO, lauryl maltose neopentyl glycol, Fos-10, Fos-12, and Fos-14 all extracted hTRPV3-GFP from the membrane, albeit with varying degrees of success. From these detergents, only DM, DDM, and LDAO retained the protein in a monodisperse state, as indicated by the symmetric main peak eluting between void volume and free GFP. The retention volume of the peak suggests that hTRPV3-GFP eluted as a tetramer. From the latter three detergents, DDM reached the highest extraction efficiency (*i.e.* the highest peak amplitude) and was therefore selected as the detergent to pursue purification of hTRPV3.

**FIGURE 2.**
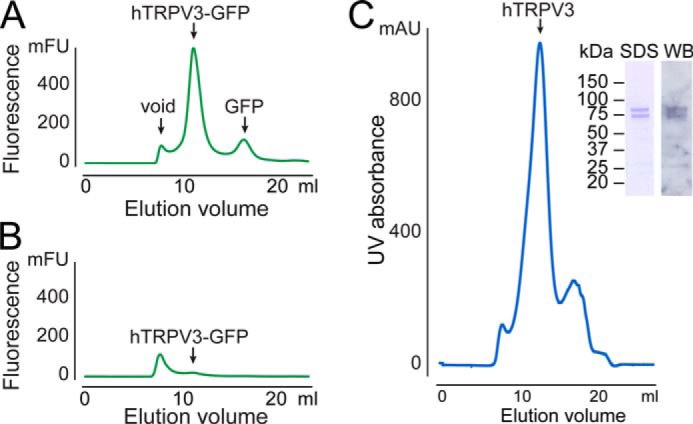
**Purification of human TRPV3.** *A*, FSEC profile of solubilized Sf9 membranes expressing hTRPV3-GFP (analytical sample taken before incubation with GFP trap). *mFU,* millifluorescence units. *B*, FSEC profile of analytical sample from the flow-through from the same GFP trap. *C*, UV detection SEC profile of hTRPV3 after elution from the GFP trap. *Inset*, Coomassie-stained SDS gel and Western blot (*WB*) of the hTRPV3 peak fraction, carried out with goat polyclonal anti-TRPV3 IgG. *mAU*, milliabsorbance units.

We then designed a two-step purification method consisting of an immunoaffinity step (GFP trap), followed by SEC. In the GFP trap (ChromoTek), GFP-directed nanobodies coupled to agarose beads specifically capture the GFP fusion protein. Fig. 2 shows FSEC profiles of solubilized crude membranes before incubation with the GFP trap (Fig. 2*A*) and from flow-through of the GFP trap (Fig. 2*B*). The hTRPV3-GFP peak present in membrane solubilizate was completely absent in flow-through, illustrating the high binding efficiency of the trap. For elution, hTRPV3 was cleaved from its GFP tag with PreScission protease. Then the eluted hTRPV3 was injected on a size exclusion column, where it eluted as a single sharp peak (Fig. 2*C*). MS analysis of this eluted peak fraction demonstrated the presence of higher masses compared with the theoretical mass of hTRPV3 (90.6-kDa monomer). This discrepancy is still unclear but could be explained by post-translational modifications inherent to the insect cell expression system. To confirm the identity of the components of the peak fraction, SDS-polyacrylamide gel analysis was done, which showed two major bands, corresponding to a slightly lower apparent mass than the theoretical mass of hTRPV3 (see Fig. 2*C*). This mass difference could be explained by an altered migration of membrane proteins, as compared with water-soluble proteins. Therefore, an MS-MS approach was performed to identify the protein content of both bands. The applied method was based on an in-gel digestion with trypsin and extraction of the proteolytic peptides followed by MS-MS analysis on a MALDI-TOF-TOF instrument (Applied Biosystems 4800 MALDI-TOF-TOF). The analysis confirmed the identity of hTRPV3 as a single protein in the bands, with Mascot scores of 44–59. Western blot of the two bands with goat polyclonal anti-TRPV3 IgG (Santa Cruz Biotechnology) further confirmed this identification (see Fig. 2*C*). The average yield for this purification protocol was ∼0.5 mg of protein/liter of Sf9 culture.

**FIGURE 3.**
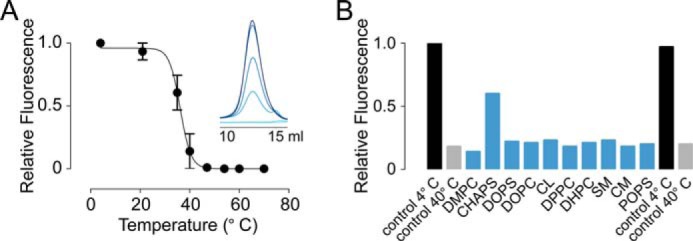
**Thermostability-based FSEC screening for hTRPV3-stabilizing additives.** *A*, melting curve of hTRPV3-GFP, yielding a melting temperature (*i.e.* the temperature at half-maximal fluorescence amplitude) of 36.2 ± 1.0 °C. Relative fluorescence is calculated from oligomeric peak amplitudes in consecutive FSEC runs with hTRPV3-GFP samples, preheated at different temperatures. Data represent mean ± S.E. (*error bars*) (*n* = 3). *Inset*, overlay of the oligomeric peak FSEC profiles. *B*, bar diagram shows relative fluorescence of oligomeric peak amplitudes from consecutive FSEC runs with a selection of additives tested for possible stabilizing effects on hTRPV3-GFP. *Black* and *gray bars* represent relative FSEC peak amplitudes of hTRPV3-GFP without additives, incubated at 4 and 40 °C, respectively (with control 4 °C set as 1). *Blue bars*, relative peak amplitudes of hTRPV3-GFP samples supplemented with various additives before incubation at 40 °C.

We then used the FSEC thermostability approach developed by Hattori *et al.* (39) to screen for additives that favor membrane protein stability in an aqueous buffer. In this assay, samples of solubilized membrane protein are heated in the presence and absence of potential stabilizing agents, such as lipids, ligands, or detergents. First, we analyzed hTRPV3 FSEC profiles over a range of temperatures and found a melting temperature of 36.1 ± 1.0 °C (Fig. 3*A*). Next, we screened over 30 different additives (see “Experimental Procedures”) and found one compound that clearly benefits hTRPV3 thermostability. Preincubation of DDM-solubilized hTRPV3 with the steroid-derived detergent CHAPS roughly tripled the fluorescence peak amplitude of heated sample (Fig. 3*B*). This result is remarkable, because CHAPS was found to be unable to extract hTRPV3 in the detergent screen but now clearly exerted a stabilizing effect on DDM-solubilized hTRPV3. We then tested a range of CHAPS concentrations (0.1–1% (w/v)) and found that 0.2% (w/v) is the optimal concentration for thermostability (CMC_CHAPS_ = 0.49% (w/v)). We therefore added CHAPS (0.2% (w/v)) to DDM in the purification of hTRPV3 for crystallization trials. Preliminary crystallization trials yielded dozens of crystals in various crystallization conditions. Unfortunately, none of these crystals exhibited satisfactory diffraction of x-ray light for structure determination. Further efforts to improve diffraction quality, including protein engineering, relipidation, and *in meso* crystallization are currently ongoing.

##### Reconstitution and Patch Clamp Experiments

**FIGURE 4.**
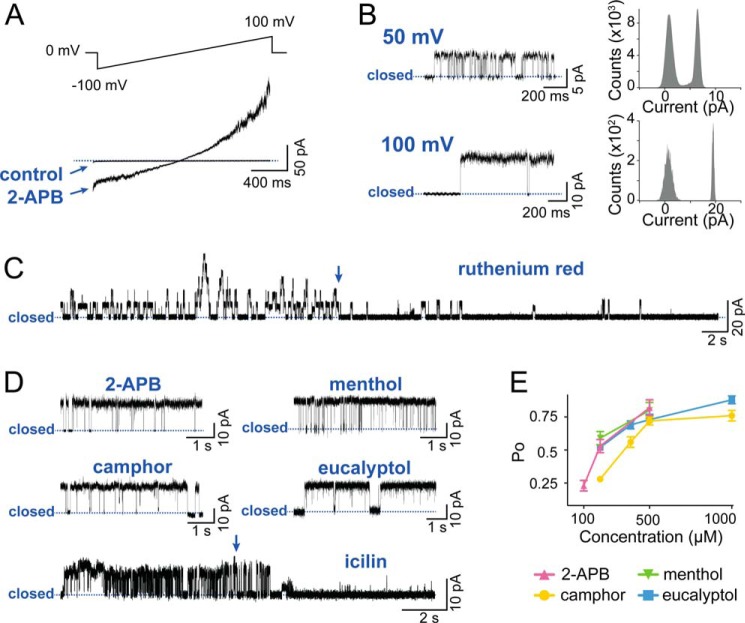
**Functional reconstitution of purified hTRPV3.** *A*, representative current recording from a planar lipid bilayer containing a high number of hTRPV3 channels during a ramp voltage protocol from −100 to 100 mV. *Arrows*, traces before (control) and after the addition of 100 μm 2-APB. *Dashed line*, zero current level. *B*, representative current traces of single-channel activity recorded from a planar lipid bilayer clamped at 50 and 100 mV after the addition of 100 μm 2-APB (*left*). The closed channel current level is indicated by *dashed lines*. Shown are corresponding current amplitude histograms (*right*), yielding a single-channel conductance of 174 pS. *C*, representative current trace from a lipid bilayer containing multiple hTRPV3 channels recorded at 100 mV. The channels were activated by 100 μm 2-APB and subsequently inhibited by 10 μm ruthenium red. The *arrow* indicates where ruthenium red was added. *D*, representative current traces of single-channel activity recorded at 100 mV in the presence of 100 μm 2-APB, 200 μm menthol, 500 μm camphor, or 500 μm eucalyptol. The *bottom trace* shows inhibition of 2-APB-activated current by 10 μm icilin. The *arrow* indicates where icilin was added. *E*, scatter plot showing open probability (*P_o_*) *versus* applied concentration of the tested agonists. *Error bars*, S.E.

Although the SEC profile provided strong indications that hTRPV3 elutes as a monodisperse tetrameric protein, it is essential to determine whether detergent-purified hTRPV3 retained its functional integrity. We therefore reconstituted purified protein in planar lipid bilayers (diphyntanoylphosphatidylcholine + 10% cholesterol) and performed patch clamp experiments. Fig. 4*A* shows a ramp voltage protocol (−100 to 100 mV in 2 s) applied on a bilayer containing a high number of inserted proteins. Current responses after the addition of 100 μm 2-APB had a reversal potential close to zero (−1.52 ± 0.03 mV, *n* = 3). We then reduced the amount of purified hTRPV3 added to the bilayers to record single-channel openings. Fig. 4*B* shows representative single-channel recordings at a constant voltage of 50 and 100 mV in the presence of 100 μm 2-APB and their corresponding current amplitude histograms. The histograms were fitted with Gaussian curves, yielding mean single-channel current amplitudes of 8.4 ± 0.7 and 17.9 ± 0.2 pA at 50 and 100 mV, respectively, which corresponds to a single-channel conductance of 174 pS. Importantly, the addition of the TRPV channel inhibitor ruthenium red to 2-APB-activated channels drastically reduced channel opening, providing evidence that the ligand-induced current is indeed the result of reconstituted TRPV3 (Fig. 4*C*). Then we tested a variety of known TRPV3 ligands to explore possible alterations in sensitivity of the detergent-purified channel. Fig. 4*D* shows representative current traces of hTRPV3 activation in bilayers by 2-APB (100 μm), menthol (200 μm), camphor (500 μm), and eucalyptol (500 μm) and inhibition of 2-APB-evoked current by icilin (10 μm). These ligands all elicited the expected functional effects on reconstituted hTRPV3. Control experiments on bilayers without hTRPV3 showed that neither ligand nor DMSO induced artifacts on the bilayers itself. To monitor the concentration range in which the agonists activate the channel, we plotted the open probability (*P_o_*) against applied concentration (see Fig. 4*E*).

##### Quenching of Intrinsic Tryptophan Fluorescence

**FIGURE 5.**
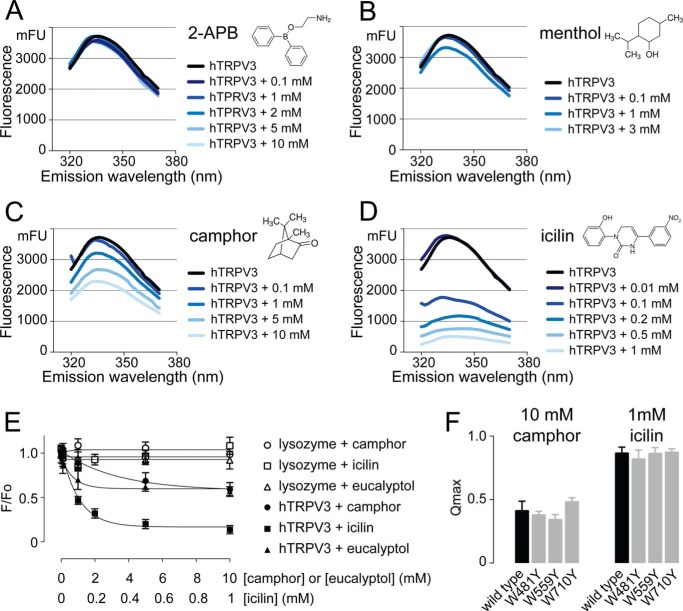
**Quenching of intrinsic Trp fluorescence by TRPV3 ligands.** The graphs show averaged fluorescence emission spectra (*n* = 3) of hTRPV3 in the presence of increasing concentrations 2-APB (*A*), menthol (*B*), camphor (*C*), and icilin (*D*). *E*, quenching plots of hTRPV3 and lysozyme in the presence of camphor, eucalyptol, and icilin show that the hTRPV3 quenching by these compounds is specific. *F*, bar diagram compares the quenching of wild type hTRPV3 with Trp mutants, recorded in the presence of a saturating concentration of camphor (10 mm) and icilin (1 mm). All data represent mean ± S.E. (*error bars*) (*n* = 3). None of the tested mutants exhibits a significant difference from wild type hTRPV3 in *Q*_max_ value (*p* > 0.05). *mFU*, millifluorescence units.

**FIGURE 6.**
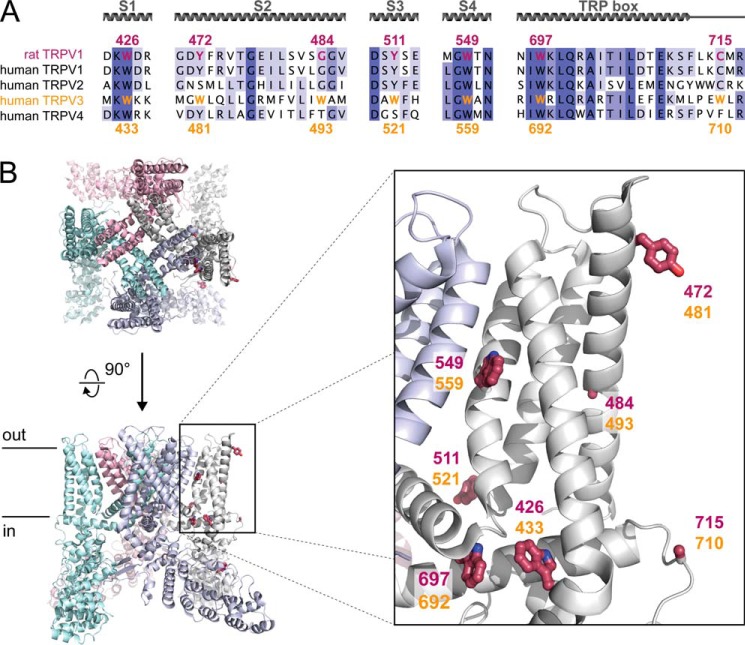
**Conservation and spatial location of Trp residues in TRPV channels.** *A*, sequence alignment of the human thermosensitive TRPV channels. Rat TRPV1 (rTRPV1) is included for comparison with the cryo-EM structure in *B*. Trp residues in the transmembrane region of hTRPV3 are *colored yellow*, and homologous positions in rTRPV1 are shown in *red. B*, schematic representations of the rat TRPV1 cryo-EM structure (32), seen along the 4-fold symmetric axis (*top left*) and in a *side view* (*bottom left*). *Ball-and-stick representations* depict rTRPV1 residues, homologous to Trp residues in hTRPV3. Numbers corresponding to Trp residues in hTRPV3 are shown in *yellow*, and numbers corresponding to residues in homologous positions in rTRPV1 are shown in *red*.

Human TRPV3 harbors 11 Trp residues in its entire sequence. These Trp residues enable hTRPV3 to fluoresce upon excitation with UV light (excitation at 295 nm; emission peak at 336 nm). We recorded intrinsic fluorescence spectra of fresh detergent-purified hTRPV3 in the absence and presence of various TRPV3 ligands (Fig. 5, *A–D*). Whereas the application of 2-APB or menthol did not alter the intrinsic fluorescence of hTRPV3, increasing concentrations of camphor, eucalyptol, or icilin progressively quenched the fluorescent signal. At saturating concentrations, the quenched fraction (*Q*_max_) was 41 ± 8% for camphor and 40 ± 2% for eucalyptol, whereas icilin quenched hTRPV3 almost to completion, with a *Q*_max_ of 86 ± 3%. Quenching plots were constructed and fitted, yielding dissociation constants (*K_D_*) of 4.78 ± 1.38 mm for camphor, 0.28 ± 0.09 mm for eucalyptol, and 0.09 ± 0.02 mm for icilin. This quenching could be caused by ligand binding, ligand-induced degradation of the protein, or a combination of both. To investigate a possible scenario of ligand-induced degradation, we preincubated hTRPV3 with saturating concentrations of camphor (10 mm) or icilin (1 mm) and analyzed protein stability on size exclusion chromatography and SDS-PAGE. For both ligands, no significant difference was observed between pretreated hTRPV3 and control, neither in quantity nor in monodispersity of the sample, ruling out ligand-induced degradation. We then evaluated potential nonspecific quenching by camphor, eucalyptol, and icilin using lysozyme as a control (Fig. 5*E*). No alteration of lysozyme intrinsic fluorescence was observed, indicating that the quenching of hTRPV3 by these ligands is specific. Next, we set out to identify individual Trp residues involved in hTRPV3 quenching by camphor and icilin. We introduced single point mutations at seven different Trp residues across the transmembrane region of hTRPV3: W433Y, W481Y, W493Y, W521Y, W559Y, W692Y, and W710Y (see Fig. 6). Due to defective expression or poor yield, we were only able to purify and examine mutants W481Y, W559Y, and W710Y. None of these mutants exhibited a significant difference (*p* > 0.05) in maximal quenched fraction (*Q*_max_) or in *K_D_* from wild type hTRPV3, indicating that Trp^481^, Trp^559^, and Trp^710^ in hTRPV3 are not involved in the quenching caused by camphor and icilin (see Fig. 5*F*). This result also implies that the quenching-sensitive Trp residues are probably among the mutations that were not tolerated.

## DISCUSSION

Structural studies of eukaryotic membrane proteins are very challenging. This is mainly due to the generally low expression levels and unstable behavior of these amphiphilic proteins after membrane extraction and purification into aqueous buffers. Here, we describe a new efficient protocol for biochemical purification of human TRPV3 that utilizes a C-terminal GFP fusion. The GFP fusion serves different purposes throughout the purification pipeline: (i) during expression, green fluorescence indicates that GFP is properly folded, which implies proper folding of the TRP channel on the N-terminal side of the fusion protein; (ii) during the detergent screen and purification process, the GFP fusion allows sensitive and selective monitoring of the protein of interest and rapid evaluation of the monodispersity on analytical samples of non-purified material on FSEC; (iii) GFP is used as a purification tag in the immunoaffinity step of purification, allowing high binding specificity that results in high sample purity. In this way, the purification protocol described here can be employed as an extension of the FSEC precrystallization screening strategy published by Kawate and Gouaux (38).

A recent study by Kol *et al.* (40) reported the purification and functional reconstitution of human TRPV3 expressed in *Escherichia coli*. Despite fundamental differences in protein expression and processing between human and prokaryote cells, they succeeded in obtaining high yields of functionally active protein. The biochemical properties of the protein, however, seem to differ significantly from our study, where human TRPV3 is expressed in a eukaryote host system. A striking difference is the inability of DDM to extract and solubilize hTRPV3 expressed in bacteria. DDM is one of the most commonly used detergents in membrane protein chemistry, and our detergent screen indicates that this detergent yields the best results out of 11 tested detergents. By contrast, the prokaryote-expressed hTRPV3 was solubilized with Fos-12, which, in our study, results in breakdown of the tetrameric hTRPV3 into multiple oligomeric states, as evidenced by the multiple peaks in the FSEC profile. Moreover, native PAGE analysis indicated that hTRPV3 purification with Fos-12 results in the formation of dimers and tetramers in solution and that both species are in equilibrium (40). Although the insect cell expression has a significantly lower yield compared with a bacterial host system (∼0.5 *versus* ∼1.2 mg/liter of culture) (40), it is still more than adequate to provide milligram quantities of purified protein necessary for crystallization purposes. Moreover, the efficient two-step purification protocol presented here yields monodisperse protein retaining functional integrity. Using planar patch clamp, we recorded 2-APB-induced currents from reconstituted hTRPV3 in lipid bilayers, with a single-channel conductance of 174 pS. This value fits very well in the range (147–197 pS) from previous reports of hTRPV3 expressed in CHO-K1 and HEK293 cells (4, 34). Other thermo-TRP channels have been successfully reconstituted into artificial bilayers, yielding crucial functional insights. This way, TRPV1, TRPM8, and TRPA1 were shown to be intrinsically sensitive to temperature and chemical stimuli (41–43). The availability of functional, detergent-purified hTRPV3 now offers opportunities to further expand our current knowledge on gating and modulation of thermo-TRPs.

So far, preliminary crystallization trials with hTRPV3 have only yielded crystals with unsatisfactory diffraction of x-ray light for structure determination. It is possible that failure to obtain well diffracting crystals is in part due to the purification protocol used. For instance, possible post-translational modifications inherent to the insect expression host or enzymatic removal of the C-terminal GFP fusion might contribute to sample heterogeneity. Another possible cause for poor diffraction of x-ray light is the presence of flexible or disordered regions in the protein. Even small differences in the primary structure can make the difference between a protein that will crystallize well and one that will not (44). Our future crystallization efforts will therefore focus on protein engineering to find minimal functional hTRPV3 constructs with as few flexible regions as possible, much alike the minimal functional constructs designed for the cryo-EM structures of rat TRPV1 (32).

We employed detergent-purified hTRPV3 in fluorescence spectroscopy measurements. Fluorescence methods can be used to study protein conformation and ligand binding through quenching of the intrinsic protein fluorescence (45, 46). This intrinsic fluorescence is mainly derived from tryptophan side chains in the protein, which act as fluorophores (47). Because the optical activity of these fluorophores is strongly influenced by their local environment in the protein, they make very useful probes to study macromolecular conformation, yielding unique information that is not available from other functional (*e.g.* electrophysiological) data. We used Trp fluorescence spectroscopy to study the interaction of detergent-purified hTRPV3 with five different modulators: activators 2-APB, camphor, eucalyptol, and menthol and the inhibitor icilin. Whereas 2-APB and menthol did not alter the intrinsic fluorescence of hTRPV3, camphor and eucalyptol caused strong quenching (∼40% quenched at saturating concentrations), and icilin quenched the fluorescence almost to completion. The dissociation constant (*K_D_*) by which camphor quenches hTRPV3 fluorescence (4.78 ± 1.38 mm) fits well in the range of reported camphor EC_50_ values on transiently expressed TRPV3 (2–7 mm) (19, 48). Unfortunately, no EC_50_ data are available for eucalyptol activation of TRPV3. In the case of icilin, our measured *K_D_* value is 1 order of magnitude higher than the reported IC_50_ value (0.09 ± 0.02 mm *versus* 7 ± 2 μm) (22). However, in this case, comparison is somewhat complicated. First, the reported IC_50_ describes the inhibition of 2-APB-evoked TRPV3 currents by icilin, whereas the *K_D_* value represents icilin quenching in the absence of 2-APB or any other agonist. Moreover, the reported IC_50_ value was established in mouse TRPV3, whereas we determined the *K_D_* value in human TRPV3 (22). In the same paper, the authors also tested human TRPV3 and found that significantly higher icilin concentrations were required for current inhibition, compared with the mouse ortholog. Although a concentration-response relationship was not determined for human TRPV3, the lower apparent sensitivity for icilin indicates that the IC_50_ on human TRPV3 may be closer to our *K_D_* value than the IC_50_ on mouse TRPV3 is.

To investigate whether the lack of quenching by menthol and 2-APB could be due to a diminished sensitivity of the purified protein, we characterized these ligands on reconstituted hTRPV3 in bilayers. All ligands produced the expected functional effects in the concentration range from observations in transiently expressed TRPV3 (21, 22, 34, 48, 49). Next, we excluded a scenario of ligand-induced degradation contributing to the quenching induced by camphor, eucalyptol, or icilin and demonstrated the specific character of the observed quenching using lysozyme as a control. Moreover, the lysozyme intrinsic fluorescence was not altered in the presence of camphor, eucalyptol, or icilin, indicating that these ligands do not absorb light at the excitation or emission wavelengths. Together, these controls lead us to conclude that the quenching occurs either as a direct result (Trp residues in or near the ligand binding site) or an indirect result (through conformational change) of ligand binding.

To dissect the observed quenching into contributions from individual Trp residues, we point-mutated tryptophan into tyrosine residues. Such a mutation retains the approximate size and aromatic character of the residue but eliminates fluorescence at the measured wavelength. Because several mutational studies found the pore region, transmembrane (TM) domain, and TRP domain to be involved in the interaction of icilin and terpenoids with TRP channels (50–53), we focused our attention on the seven tryptophan chains residing in this part of hTRPV3 (see Fig. 6). Due to defective expression or low yield, we were only able to analyze W481Y, W559Y, and W710Y for quenching by camphor or icilin. The maximal quenched fraction and dissociation constants of all three mutants were similar to those for wild type hTRPV3, indicating that Trp^481^, Trp^559^, and Trp^710^ are not involved in the quenching by camphor and icilin. A simple but clear conclusion is that neither of these residues are directly involved in binding of camphor and icilin and that these positions are not affected by ligand-induced conformational changes.

Our data illustrate that 2-APB and camphor exert distinct mechanisms of action on hTRPV3. This is supported by a mutation study that showed that two residues in mouse TRPV3 (His^426^ and Arg^696^) are crucial for sensitivity to 2-APB but not to camphor (48). It was also reported that long term (5–15 min) incubation with terpenoid ligands like camphor, leads to desensitization of TRPV3, whereas 2-APB sensitizes channel activity (35).

Despite their structural relatedness, camphor and menthol display a remarkable difference in quenching behavior. Camphor was previously shown to interact with a conserved cysteine residue (Cys^619^) in the pore region of mouse TRPV3 (53). Mutation of this residue resulted in total loss of camphor sensitivity, whereas responses to 2-APB and dihydrocarvacrol remained untouched. Unfortunately, the recently published cryo-EM structures of rat TRPV1 depict a minimal functional construct in which, among others, pore region residues 604–626 are deleted (32). This makes it difficult to imagine the exact spatial location of Cys^619^, but if this residue does indeed take part in a camphor binding site, this site would be distant from any Trp residue in TRPV3. However, camphor clearly quenches hTRPV3 tryptophan fluorescence in our study. This suggests that the quenching by camphor is a result of an indirect (due to a conformational change) rather than a direct effect (ligand binding). The complete absence of quenching by menthol, on the other hand, does not necessarily imply that menthol occupies a different binding site on hTRPV3 than camphor. Because of their structural relation, it is conceivable that menthol would bind to a similar site on hTRPV3. In a functional study, TRPV3 agonists were classified into three groups, based on their pharmacological profile (35). A first category includes agonists like 2-APB that do not induce desensitization of TRPV3. The two other categories consist of agonists that cause different types of desensitization upon long term exposure. The bicyclic terpenoids (*e.g.* camphor and eucalyptol) induce acute desensitization, whereas the monocyclic terpenoids (*e.g.* menthol and dihydrocarveol) cause tachyphylaxis. Keeping a conformational change at the basis of camphor and eucalyptol quenching in mind, the difference in quenching between camphor/eucalyptol and menthol could illustrate the distinct functional behavior of the two classes of terpenoids. Indeed, during the fluorescence recordings, protein samples were incubated with ligands on a time scale that would allow desensitization to occur (>5 min). It appears that quenching may be associated with negative functional effects (*i.e.* inhibition by icilin and desensitization by camphor and eucalyptol).

A remarkable observation is the extensive quenching exercised by icilin, which almost completely extinguished hTRPV3 fluorescence. This dramatic change indicates major structural rearrangements in TRPV3 upon icilin binding. Icilin sensitivity in TRPM8 is thought to be mediated through residues in the intracellular loop connecting TM2 and TM3 (50). These residues correspond to an analogous region in TRPV1 believed to be important for capsaicin sensitivity (54). Similarly, residues in TM2, TM3, and TM4 were pointed out as crucial determinants for menthol activation of TRPM8 and for activation of TRPV4 by 4α-phorbol esters (51, 52, 55). It was proposed that menthol possibly intercalates between TM2 and TM4 to activate TRPM8. In a more general mechanism, TRP channel ligands were proposed to shift the voltage dependence of channel activation through interaction with TM1–TM4 (52). Although it does not reveal information about a possible binding site, the difference in quenching between menthol and icilin on hTRPV3 might reflect the inverse functional effect of icilin on this channel. In another model, ligand binding is mechanically coupled to channel opening through the TRP domain, which is involved in translating the initial ligand-binding event to the allosteric conformational changes that cause channel opening (51). Such a model would be less evident in our data, because mutation of Trp^710^, which is adjacent to the TRP domain, does not change quenching in hTRPV3.

In conclusion, our data confirm and expand the hypothesis that TRPV ion channels are allosterically modulated by different chemical ligands through independent and distinct molecular mechanisms. It is, however, clear that high-resolution structures will be required to validate and establish ligand binding sites and to help unravel the mechanisms that underlie gating in TRP channels. The TRPV3 purification protocol presented here provides a crucial step toward high-resolution structure elucidation.

## Footnotes

2 The abbreviations used are:

TRP: transient receptor potential
2-APB: 2-aminoethoxydiphenyl borate
DDM: dodecyl maltoside
DM: decyl maltoside
Fos-10: -12, and -14, decyl-, dodecyl-, and tetradecylphosphocholine, respectively
SEC: size exclusion chromatography
FSEC: fluorescence detection SEC
TRPV: transient receptor potential vanilloid
hTRPV3: human TRPV3
LDAO: lauryl dimethylamine oxide
TM: transmembrane
TRPA: TRP ankyrin
TRPM: TRP melastatin
CHAPSO: 3-[(3-cholamidopropyl)dimethylammonio]-2-hydroxy-1-propane sulfonic acid
CMC: critical micelle concentration
pS: picosiemens.
